# Levamisole/Cocaine Induced Systemic Vasculitis and Immune Complex Glomerulonephritis

**DOI:** 10.1155/2015/372413

**Published:** 2015-07-28

**Authors:** Lohit Garg, Sagar Gupta, Abhishek Swami, Ping Zhang

**Affiliations:** ^1^Department of Internal Medicine, Oakland University William Beaumont School of Medicine, Royal Oak, MI 48073, USA; ^2^Department of Nephrology, Washington University in St. Louis, St. Louis, MO 63130, USA; ^3^Department of Nephrology, Oakland University William Beaumont School of Medicine, Royal Oak, MI 48073, USA; ^4^Department of Pathology, William Beaumont Hospital, Royal Oak, MI 48073, USA

## Abstract

Levamisole is an antihelminthic and immunomodulator medication that was banned by the USFDA in 1998. It has been increasingly used to adulterate cocaine due to its psychotropic effects and morphological properties. Adverse reactions including cutaneous vasculitis, thrombocytopenia, and agranulocytosis have been well described. Despite systemic vasculitis in this setting, renal involvement is uncommon. We report here a case of ANCA positive systemic vasculitis with biopsy proven immune complex mediated glomerulonephritis likely secondary to levamisole/cocaine. A 40-year-old Caucasian male with no past medical history presented with 3-week history of fatigue, skin rash, joint pains, painful oral lesions, oliguria, hematuria, worsening dyspnea on exertion, and progressive lower extremity edema. He had a history of regular tobacco and cocaine use. Lab testing revealed severe anemia, marked azotemia, deranged electrolytes, and 4.7 gm proteinuria. Rheumatologic testing revealed hypocomplementemia, borderline ANA, myeloperoxidase antibody, and positive atypical p-ANCA. Infectious and other autoimmune workup was negative. Kidney biopsy was consistent with immune mediated glomerulonephritis and showed mesangial proliferation and immune complex deposition consisting of IgG, IgM, and complement. High dose corticosteroids and discontinuing cocaine use resulted in marked improvement in rash, mucocutaneous lesions, and arthritis. There was no renal recovery and he remained hemodialysis dependent.

## 1. Introduction

Levamisole is an antihelminthic and immunomodulator medication previously used to treat steroid resistant nephrotic syndrome in pediatric population and also as adjuvant chemotherapy for colorectal and breast cancer [1, 2]. It was banned by USFDA in 1998 due to serious side effects including nonspecific rash, thrombocytopenia, and agranulocytosis. It was associated with reversible cutaneous vasculitis with earliest cases reported in 1970s [3, 4]. Particularly striking feature in these cases was purpura involving the ear. More recently, it has increasingly been used as a cutting agent in cocaine especially in the United States. Nearly 69% of cocaine samples seized by the Drug Enforcement Administration (DEA) in 2008-2009 tested positive for adulteration [5]. Renal involvement in the form of glomerulonephritis is relatively uncommon. We describe here a case of ANCA positive systemic vasculitis with biopsy proven immune complex mediated glomerulonephritis secondary to levamisole/cocaine, a rare entity.

## 2. Case Presentation

A 40-year-old Caucasian male with no past medical history presented to the emergency room with one-week history of progressive shortness of breath on exertion. He also complained of palpitations, fatigue, and orthopnea. In addition, he complained of progressive lower extremity swelling for the last 3 weeks and multiple painful ulcerations on his tongue and in his mouth for 2 weeks. History was also notable for multiple joint pains for 6 months. He was diagnosed with Lyme's disease and was treated with high dose doxycycline for 2 months. Two months prior to admission, he noticed diffuse nonitchy rash on his chest, back, abdomen, arms, and legs that subsequently resolved. One month prior to admission, he noticed decreased urine output and dark colored urine. There was no history of fever, chills, weight loss, night sweats, cough, chest pain, or hemoptysis. He denied having any dry eyes, oral ulcers, photosensitivity, abdominal pain, hematuria, dysuria, or neurologic symptoms.

Medications included doxycycline and ibuprofen. He had history of long standing tobacco abuse, alcohol use, and regular cocaine use. He denied having any tattoos, sick contacts, recent travel, or environmental or occupational exposure.

On examination, he was afebrile, tachycardic, tachypneic, and hypoxic on room air. The tongue had hyperkeratotic, hyperpigmented papules. There were scattered erythematous maculopapular lesions on the chest. He had bilateral lower extremity edema with skin changes suggestive of chronic venous stasis and prominent symmetric synovitis of metacarpophalangeal and wrist joints. Chest auscultation revealed diffuse rales bilaterally. Cardiovascular, abdominal, and neurologic examinations were unremarkable.

Lab results are shown in Table 1. Notable lab abnormalities included anemia and severe azotemia with multiple electrolyte abnormalities (no records of prior serum creatinine values). Urinalysis showed significant hematuria and proteinuria. Urine protein/creatinine ratio was 4.7. Acute phase reactants ESR and CRP were elevated. BNP and PTH were also elevated. Rheumatologic testing revealed borderline ANA, positive atypical p-ANCA (1 : 640), and positive anti-myeloperoxidase antibodies. Complement levels (C3 and C4) were low. Remainder of the rheumatologic workup was negative. Chest X-ray showed pulmonary edema. Urine screen for drugs returned positive for cocaine and levamisole. Unfortunately quantification of levamisole could not be performed on time and resulted negative.

**Figure 1:**
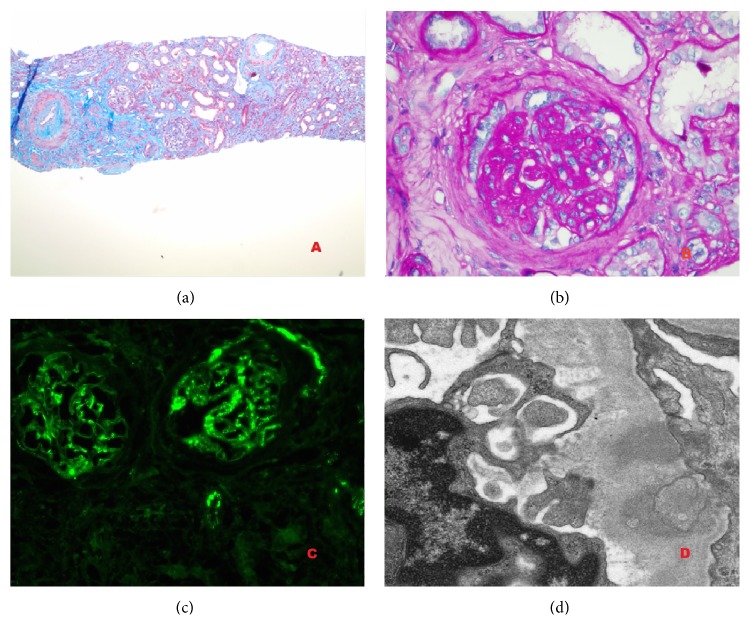
(a) Masson trichrome stain (100x) revealed severe interstitial fibrosis, thickened arterioles, and mild proliferation of glomeruli. (b) PAS stain (600x) showed mild mesangial proliferation and segmental sclerosis. No extra capillary crescent or necrosis was identified. Six of 10 glomeruli were globally sclerosed. (c) IF showed 2-3+ IgG and C3 deposit mainly in mesangium and also along the capillary loops. (d) Electron microscopy showed intramembranous and subepithelial electron dense deposits with occasional subendothelial and mesangial deposits. There were segmental foot process effacement and focal mesangial interposition.

Kidney biopsy showed diffuse tubulointerstitial fibrosis with the majority of glomeruli globally sclerosed. Few intact glomeruli showed mesangial proliferation and immune complex deposition consisting of IgG, IgM, and complement in mesangial and endocapillary distribution. It was consistent with immune mediated glomerulonephritis (Figure 1). Skin biopsy of the rash was consistent with leukocytoclastic vasculitis.

Given the clinical, laboratory, and pathologic findings, we concluded that the ANCA associated systemic vasculitis and immune complex mediated glomerulonephritis were secondary to levamisole/cocaine use.

## 3. Clinical Course

He required mechanical ventilation for acute hypoxic and hypercarbic respiratory failure. He was placed on continuous renal replacement therapy for severe azotemia with multiple electrolyte abnormalities including hyperkalemia. He was started on high dose steroids with marked improvement in his rash, mucocutaneous lesions, and arthritis. There was no renal recovery and he remained hemodialysis dependent. He was discharged on prednisone 40 mg daily, slowly tapered, and stopped after 3 months with resolution of arthritis and skin rash. Repeat rheumatologic workup was negative for ANCA after 3 months of steroid therapy and cocaine abstinence. He is currently undergoing intermittent hemodialysis and is awaiting renal transplant.

## 4. Discussion

Levamisole contamination of cocaine has become a widespread health problem. In 2009, nearly two-thirds of cocaine samples seized by the DEA in the US [5] were found to be contaminated with levamisole. It is thought to potentiate the psychotropic effect of the illicit drug by increasing dopamine in the brain, acts as a bulking agent, and is morphologically difficult to recognize as an adulterant. It continues to gain medical attention as more and more cases of adverse effects of levamisole are being reported.

Earliest cases of levamisole induced necrotizing cutaneous vasculitis were reported in 1970s by Scheinberg et al. and Macfarlane and Bacon [3, 4]. Segal et al. reported levamisole induced arthritis in patients treated with levamisole as immunomodulator for Crohn's disease [6]. Strazzula et al. reported multiple cases of purpuric skin lesions in levamisole exposed patients that required less aggressive strategies than what is used for primary ANCA associated vasculitis [7]. Most of these patients tested positive for anticardiolipin antibodies, ANA, p-ANCA, or c-ANCA, all of which resolved after drug withdrawal.

Cocaine itself has been associated with ANCA positive cutaneous vasculitis but systemic organs are rarely affected [8, 9]. The contamination with levamisole adds an additional compounding factor and toxicity can occur with snorting, smoking, or intravenous use. Most of the affected individuals are chronic, habitual users suggesting large cumulative, dose dependent response [10–14].

First description of levamisole induced nephropathy was as early as in 1978 when Hansen et al. described a case of rheumatoid arthritis treated with levamisole developing a pruritic rash, leukopenia, thrombocytopenia, and proteinuria [15]. Kidney biopsy revealed granular mesangial deposits of IgA, IgG, IgM, and C3. Zwang et al. described a similar presentation with arthritis, neutropenia, purpuric rash, and acute kidney injury that had also urinalysis consistent with proteinuria but no red blood cells or cast [16]. Díaz et al. also reported cutaneous vasculitis, leukopenia, renal failure, and nephrotic proteinuria in their patient abusing intravenous cocaine [17]. Unfortunately no renal biopsies were performed in these patients. McGrath et al. in their case series of 30 patients with ANCA positivity associated with levamisole-contaminated cocaine use found 8 patients to have abnormal urinalysis with dipstick proteinuria, hematuria, or the presence of cellular casts on microscopy [18]. Two of these developed severe acute kidney injury and one underwent renal biopsy; however, that revealed pauci-immune focal necrotizing and crescentic glomerulonephritis. The mechanism in the pathogenesis of levamisole/cocaine induced ANCA positive systemic vasculitis and immune complex glomerulonephritis is unclear.

Renal involvement is relatively uncommon with ANCA positive vasculitis caused by levamisole/cocaine. To the best of our knowledge, this may be the second reported case with biopsy proven immune complex mediated glomerulonephritis. Rheumatologic workup in our patient was positive for atypical p-ANCA, myeloperoxidase antibody, and hypocomplementemia. In the presence of adequate exposure, these abnormalities are now increasingly recognized as very specific for levamisole-adulterated cocaine exposure [18–20].

## 5. Conclusion

This case illustrates the growing issue of cocaine abuse and levamisole contamination. Levamisole induced vasculitis is a diagnosis of exclusion but should be considered in cocaine users presenting with vasculitis, arthralgia, leukopenia, and positive ANCA titers after excluding infections and other idiopathic vasculitides. Timely recognition of this clinical entity is important to avoid misdiagnosis and unnecessary prolonged treatment with harmful cytotoxic agents as discontinuation of cocaine use could result in resolution of symptoms.

## Figures and Tables

**Table 1:** Lab results.

Variable	Result on admission (reference range)	Result at discharge (reference range)
White cell count	12.7 (3.5–10.1)	5.7 (3.5–10.1)
Neutrophils %	10.8 (1.6–7.2)	4.6 (1.6–7.2)
Lymphocytes %	1.4 (1.1–4.0)	0.8 (1.1–4.0)
Eosinophils %	0.1 (0.0–0.4)	0.0 (0.0–0.4)
Basophils %	0.1 (0.0–0.1)	0.0 (0.0–0.1)
Monocytes %	0.3 (0.0–0.9)	0.3 (0.0–0.9)
Hemoglobin, g/dL	6.1 (13.5–17.0)	9.4 (13.5–17.0)
Platelet count	334 (150–400)	267 (150–400)
Sodium mmol/L	120 (135–145)	138 (135–145)
Potassium mmol/L	6.9 (3.5–5.2)	4.6 (3.5–5.2)
Chloride mmol/L	87 (95–107)	100 (95–107)
Carbon dioxide mmol/L	10 (21–31)	24 (21–31)
Blood urea nitrogen mg/dL	195 (8–22)	67 (8–22)
Creatinine mg/dL	20.83 (0.60–1.40)^**∗**^	6.77 (0.60–1.40)
Calcium mg/dL	5.6 (8.5–10.5)	8.6 (8.5–10.5)
Phosphorus mg/dL	20.2 (2.3–4.3)	5.8 (2.3–4.3)
Aspartate aminotransferase, U/L	721 (10–37)	39 (10–37)
Alanine aminotransferase, U/L	252 (9–47)	62 (9–47)
Alkaline phosphatase, U/L	146 (30–110)	113 (30–110)
Total bilirubin, mg/dL	0.8 (0.3–1.2)	0.3 (0.3–1.2)
Albumin, g/dL	2.9 (3.5–5.1)	3.4 (3.5–5.1)
Protein, g/dL	5.3 (6.4–8.6)	5.3 (6.4–8.6)
International normalized ratio	1.7	1.1
Partial thromboplastin time, sec	44.1 (25.0–32.0)	28.8 (25.0–32.0)
Urine protein/creatinine ratio	4.7 (0.0–0.2)	
Urinalysis	3+ protein, 2+ blood, 10–20 RBC, and Hyaline and RBC cast	
ESR, mm/hr	61 (0–20)	
CRP mg/dL	7.4 (0.0–1.0)	
Complement C3, mg/dL	42 (70–176)	
Complement C4, mg/dL	7.7 (12.1–42.9)	
Anti-nuclear antibodies, IU/mL	<1 : 160 (<1 : 160)	Negative (<1 : 160)
Anti-double-stranded DNA, IU/mL	6.6 (0.0–29.9)	
Anti-neutrophil cytoplasmic antibody	1 : 640 p-ANCA (<1 : 20)	
Anti-SSA, U	0.9 (<20)	
Myeloperoxidase antibody, U	2.8 (<0.4)	
Proteinase-3 auto antibody, U	0.4 (<0.4)	
Cryoglobulin screen	Negative	
Lupus anticoagulant	Negative	
Serum and protein electrophoresis	Negative for monoclonal antibodies	
Tuberculin skin test	Negative	
HIV-1 and HIV-2 antibodies	Negative	
Acute hepatitis panel	Negative for hepatitis B and hepatitis C	
Rapid plasma reagin	Negative	
Histoplasma urine antigen	Negative	
Blood and urine cultures	Negative	

^*∗*^No serum creatinine values were available prior to admission.

## Abbreviations

ANA: Antinuclear antibody
p-ANCA: Perinuclear anti-neutrophilic cytoplasmic antibody
USFDA: US Food and Drug Administration
ESR: Erythrocyte sedimentation rate
C-RP: C-reactive protein
BNP: B-natriuretic peptide
PTH: Parathyroid hormone
Anti-MPO: Anti-myeloperoxidase antibody.
